# Comparison of First Accessory Cone Penetration after Using Stainless-Steel and Ni-Ti Spreaders in Curved Resin Blocks

**Published:** 2008-01-10

**Authors:** Nahid Mohammadzade Akhlaghi, Nasrin Roghanizad, Zohreh Khalilak, Neda Rezaie

**Affiliations:** 1 *Department of Endodontic, Dental School of Islamic Azad University and Iranian Center for Endodontic Research, Tehran, Iran*; 2 *General Practitioner, Tehran, Iran*

**Keywords:** Gutta-Percha, Nickel-Titanium, Root Canal Obturation, Stainless-Steel

## Abstract

**INTRODUCTION:** The purpose of this study was to compare first accessory gutta-percha penetration after the use of nickel-titanium (Ni-Ti) or stainless-steel spreaders in curved resin blocks.

**MATERIALS AND METHODS:** Forty resin blocks with 30º and 45º curvatures (20 blocks for each) were prepared and #30 master cones were placed in canals. In part one, under 20 Newton pressure, Ni-Ti or stainless-steel spreaders were placed alongside the master cone and their penetration was measured with a millimeter ruler. In part two, after removing the spreader, one of the accessory cones (#15, #20 or MF) was substituted in the canal and its penetration was measured. The Mann-U Whitney test was used for the final evaluation.

**RESULTS:** In both curvatures, the penetration of Ni-Ti spreaders were significantly more than stainless-steel spreaders (p<0.005). Penetration of the first accessory cones (#15 and MF) in Ni- Ti spreader group were also significantly more than stainless-steel group (p<0.05) (in both curvature groups).

**CONCLUSION:** This study showed that Ni-Ti spreaders and #15 accessory cones penetrated deeper in curved canals than stainless spreaders and #15 accessory cones. Therefore, the use of NiTi spreader in lateral condensation technique is suggested for better results.

## INTRODUCTION

Placement of endodontic spreaders alongside the master cone to the depth of 0.5 to 1 millimeter short of the working length has been advocated for optimum obturation. The accessory cones should be placed to the same length as the penetration of spreader; to obtain favorable void less obturation (1-2). Studies have shown that the lateral condensation technique provides an excellent apical seal (3-4).

However, in curved canals the results of this technique will not be predictable (5-7). This may be due to inadequate condensation of gutta-percha (GP) because of inability of stainless-steel (SS) spreaders to penetrate to within 1 mm of the working length in curved canals (8). This might be addressed to the inflexibility of stainless steel spreaders that in turn leads to an inadequate apical seal (2) and wedging effect of the spreader that may cause root fracture, if the operator attempts to force it to the proper length (9). Sobhi and Khan (10) reported significantly deeper penetration of nickel-titanium (Ni-Ti) finger spreaders than the SS spreaders. Also they showed easer penetration of NiTi finger spreader than the SS spreaders especially in curved canal. Berry *et al. *(8) showed that no statistically significant difference in penetration of two types of spreaders was found in straight canals. As the canal curvatures increased the penetration of SS spreader was decreased. But the ability of NiTi spreader to approach the apex was not affected by an increase in root canal curvature. Willson and Bumgarthner (11) reported no significant difference between Ni-Ti and SS spreader penetration using 0.02 tapered gutta- percha in small canal curvature of (0 to 20 degrees). Both Ni-Ti and SS spreaders penetrated to a greater depth as canal curvatures increased to greater than 20 degrees (11). Schmidte *et al. *(2) showed that the average depth of penetration of a Ni-Ti spreader with conjugation of a master gutta- percha cone when using a standardized 1.5 kg force was significantly greater than that for a SS spreader. But, when the differences between depth of spreader and accessory cone penetration compared; there was no significant difference between the Ni-Ti and SS spreaders. Several studies have shown that the size of the accessory cones relative to the spreader size has been a noticeable problem (12-14). To eliminate confusion in sizing, Hartwell *et al. *(12) successfully urged that spreaders be manufactured in standardized sizes.

The purpose of this study was to compare first accessory cone penetration after using Ni-Ti or SS spreaders in curved canals of resin blocks.

## MATERIALS AND METHODS

Forty resin blocks with 30º and 45º curvatures were used in this study. Canals were prepared using Passive Step Back technique (15) by K- Flexofiles (Maillefer Dentsply, Ballaigues, Switzerland).

First, canal length was determined by a #10 K file (Maillefer Dentsply, Ballaigues, Switzerland). The working length (WL) was established 1 mm shorter than the canal length. After pre-flaring by GG #2, 3 canals were prepared within WL to #30 master apical file (MAF) and flared by GG #2, 3 and step back to K-file #45 with 1 mm increments. After removing each file or GG drill, 2 mL of saline was used for irrigation. Canal patency was checked by #10 file. After finishing the canal preparation, #30 GP cone with 0.02 tapered (Diadent, Burnaby, B.C., Canada) was placed in the canal to the WL.

Resin blocks were divided into two experimental groups 30º and 45º curvatures twenty blocks of each. Each of these two groups was classified in to SS and Ni-Ti groups of ten blocks in each. In part one #25 (B/0.02) SS or Ni-Ti finger spreaders (Maillefer Densply, Ballaigues, Zwitzerland) were put alongside the master gutta-percha cone and remained for 5 sec in canals under 20 Newton (~2 kg) pressure applied by an Instron machine (Instron Corp., Canton, ME, USA). Spreader penetration was measured by a millimeter ruler calibrated in 0.5 mm increments. In part two, all of the resin blocks were used three times. Each time after removal of spreaders, one of the 0.02 tapered accessory cones #15, #20 or conventional MF accessory gutta-percha cone (Diadent, Burnaby, B.C., Canada) was put alongside its master cone and the penetration was measured in each group. The results were compared and evaluated by Mann-U Whitney test.

## RESULTS

Five resin blocks (two of 30º curvature groups and three of 45º curvature groups) were discarded because of file breakage and canal obstruction. The average working length of the canals was 16.8 mm.

**Figure 1:**
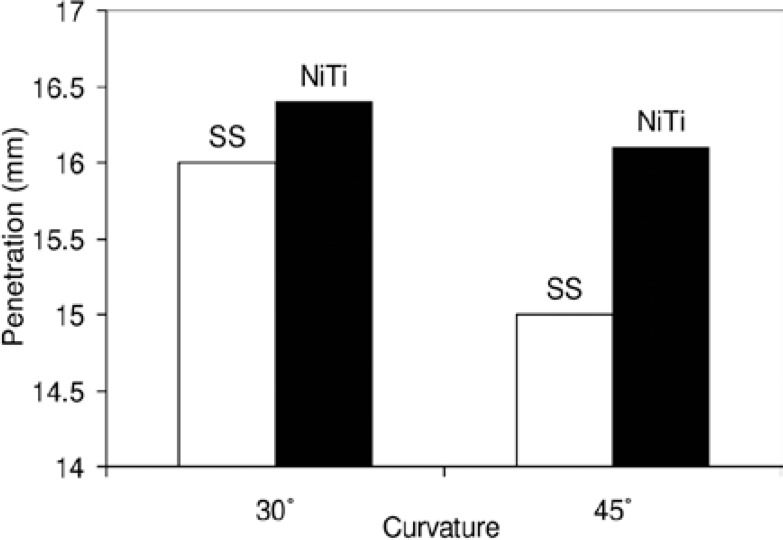
Comparison of SS / Ni-Ti spreader penetration in 30º and 45º curvatures

In part one of the study, comparison of SS and Ni-Ti finger spreader penetration alongside the master gutta-percha cone showed that in both 30º and 45º curvatures Ni-Ti spreaders penetrated significantly further than SS spreaders (p<0.005). With regard to the curvature of the canals, both spreaders' penetration was more in 30º curvatures compared with 45º curvatures. However, the difference was only significant in the SS spreader group, but not significant for the Ni-Ti spreader group (Figure 1).

Part two of the study showed accessory cone #20 penetrated less than #15 and MF accessory cones in either spreader or curved groups On the other hand #15 and MF first accessory cone penetrated significantly more in the Ni-Ti spreader group (p<0.05). Number 20 cones also penetrated farther in the Ni-Ti spreader group, but the differences were not significant (Table 1).

In the SS spreader group both #15 and MF cones penetrated significantly more in a 30º curvature compared to the 45º curvature (p<0.005). In the Ni-Ti spreader group, the penetration of the first #15 accessory cones in both curvatures was not significantly different (p>0.05).

**Table 1:** Comparison of first accessory cone penetration after using Ni-Ti/SS finger spreaders in 30º and 45º curved canals (mean ±SD in mm)

**G** **P**	**30** **º**		**45º**	
	**S** **S**	**Ni-Ti**	**S** **S**	**Ni-Ti**
15	16.1±0.5	16.4±0.5	15.6±0.4	16.1±0.4
20	15.5±0.7	15.8±0.6	15.1±0.5	15.1±0.6
MF	16.1±0.6	16.4±0.4	15.4±0.5	16.0±0.4

## DISCUSSION

According to the results of this study, penetration of Ni-Ti spreaders in both 30º and 45º curvatures was significantly more compared with SS spreaders. This is similar to the results of Sobhi and Khan (10), Schmidt *et al. *(2) and also Wilson and Baumgartner studies (11). Regardless of the degree of curvature, all of them found that Ni-Ti spreaders penetrated further into the canal.

The present study also found that Ni-Ti spreader penetration in both curvatures was similar, while SS spreader penetration was influenced by the degree of root curvature.

This result was the same as the findings of Berry *et al.*(8). Sobhi and Khan (10) also found that an increase in the canal curvature will reduce the ease of spreader penetration. However, Wilson and Baumgartner (11) showed that penetration of Ni-Ti and SS spreaders in curvatures under 20º were less than their penetration in curvatures greater than 20º. They explained that the force exerted on the canal walls by the shape memory effect of Ni-Ti-rotary files may cause more dentin removal and wider preparation in curved canals compared to straight canals.

Dulaimi and Wali (16) also concluded that space prepared by flaring the canal will affect the amount of spreader penetration. Berry *et al. *(8) reported no significant difference between SS and Ni-Ti spreader penetration in straight canals.

In the second part of the present research, it was realized that the penetration of accessory cones in the Ni-Ti spreader groups was further than SS spreader groups. This was unlike the results of Schmidt *et al. *(2) which showed no significant difference between first accessory cone penetration in both spreader groups. In their study, a #35 file was chosen as the master apical file. Canals were obturated using #30 Ni-Ti/SS spreaders and #25 accessory cones.

The size of the accessory cones relative to the spreader size has been discussed and shown in several studies to be a significant problem (12-14). Hartwell *et al. *urged that spreaders be made in standardized sizes (12).

According to the present study, accessory cone penetration in 30 º curvatures was more than in 45º curvatures. The penetration of #15 or MF accessory cones, in both spreader groups and curvatures, was further than that of #20 cones. Since the tapering of MF accessory cones are not adjusted with 0.02 tapering of hand files it seems that the use of MF accessory cones will cause early obturation of coronal part of the canal.

## CONCLUSION

This in vitro study showed that Ni-Ti spreaders and #15 accessory cones penetrated further in curved canals. Therefore, their use in the lateral condensation technique is suggested for better results.
